# Updates on the Management of Advanced, Metastatic, and Radioiodine Refractory Differentiated Thyroid Cancer

**DOI:** 10.3389/fendo.2017.00312

**Published:** 2017-11-20

**Authors:** Dario Tumino, Francesco Frasca, Kate Newbold

**Affiliations:** ^1^Clinical Oncology, Royal Marsden NHS Foundation Trust, London, United Kingdom; ^2^Endocrinology, Department of Clinical and Experimental Medicine, University of Catania, Garibaldi-Nesima Medical Center, Catania, Italy

**Keywords:** thyroid cancer, radioiodine refractory, kinase inhibitors, redifferentiation, distant metastasis

## Abstract

Differentiated thyroid cancer (DTC) accounts for 95% of all thyroid cancers and is generally an indolent tumor, treated effectively with surgery, radioactive iodine, and thyroid-stimulating hormone suppressive therapy. However, 5–10% of patients have advanced disease, with aerodigestive tract invasion, distant metastases, or radioiodine refractory disease, with poor prognosis. This review focuses on the approaches for treating advanced DTC, including management of gross extra-thyroidal extension, recurrent loco-regional or distant metastatic disease, the role of external beam radiation therapy and systemic treatment. Locally ablative treatment modalities, including surgery, radiation therapy, and thermal ablation are evolving and can be used in selected patients. In recent years, new therapeutic agents with molecular targets have become available and two multi-kinase inhibitors, Sorafenib and Lenvatinib, have been licensed for iodine refractory DTC showing an advantage in terms of progression-free survival, although an impact on overall survival has not been proven yet. Management of advanced thyroid cancer can be challenging but a multidisciplinary approach can significantly improve outcomes for this patient population.

## Introduction

**Figure 1:**
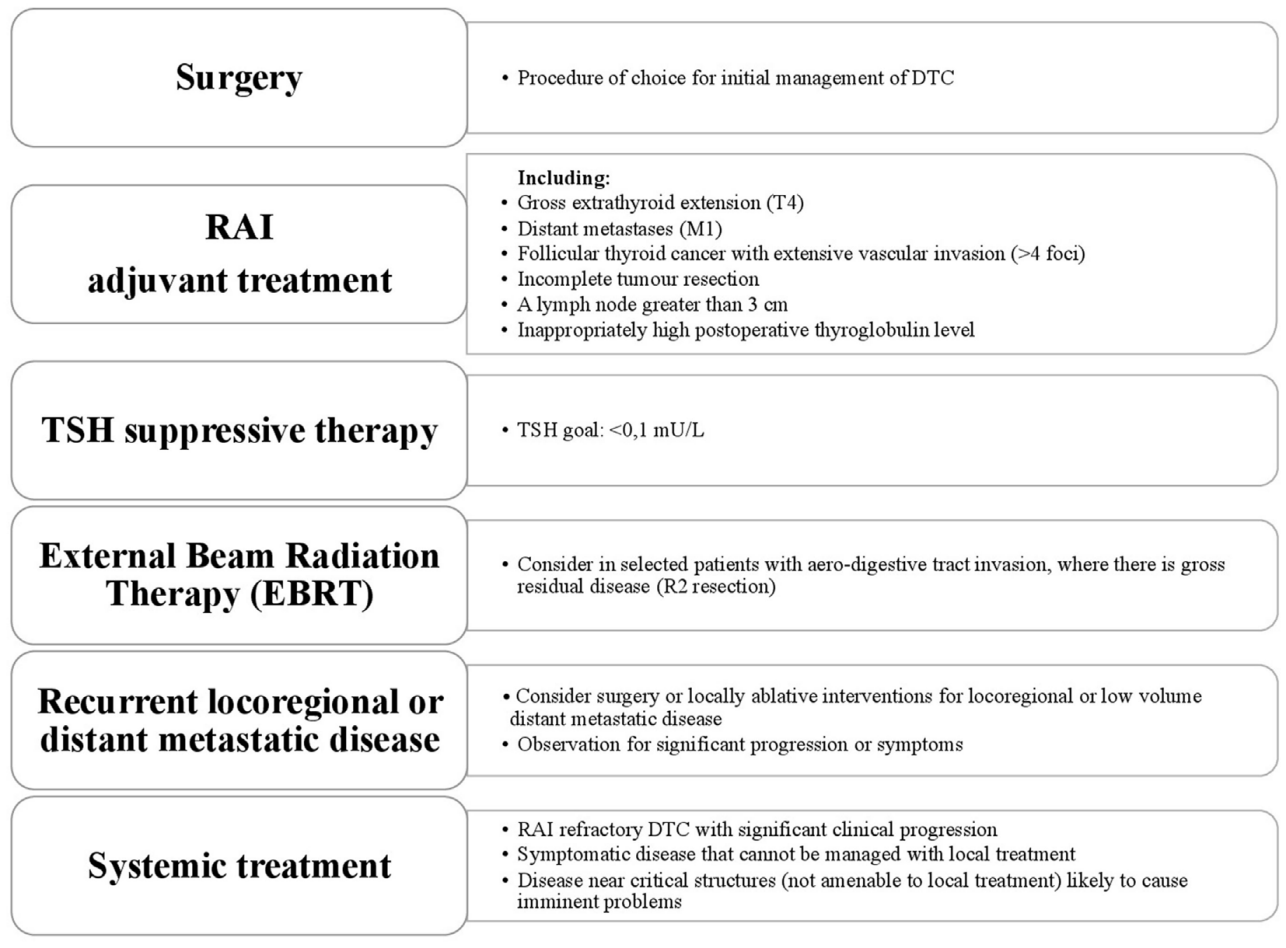
Treatment approach for a patient with advanced differentiated thyroid cancer.

Thyroid cancer has a wide range of clinical behavior from an indolent tumor with low mortality in most cases to aggressive disease. Both papillary and follicular cancers arise from thyroid follicular epithelial cells and they are grouped together under the umbrella term differentiated thyroid cancer (DTC), accounting for 95% of cases. Surgery followed by radioactive iodine or observation effects a cure in the majority (1). Approximately 5% of patients with DTC will present with locally advanced disease (2, 3). Distant metastases will develop in 10% and it is the distant metastases that are the main cause of thyroid cancer-related deaths with overall mortality rates of 65% and 75% at 5 and 10 years, respectively (4). The American Thyroid Association (ATA) Guidelines classify DTC with gross extra-thyroidal extension (ETE), distant metastases, incomplete tumor resection, inappropriately high postoperative thyroglobulin (Tg), involved lymph nodes greater than 3 cm and follicular cancer with extensive vascular invasion, as “high risk” for recurrence (5). About one-third of advanced DTC (A-DTC) have metastatic lesions with low avidity for iodine at the time of diagnosis (6). This can also occur during the progression of the disease, when the ability to concentrate radioiodine (RAI) is lost or the tumor progresses despite significant uptake of RAI. These three situations, together with a mixed picture of both RAI avid and non-avid disease, often 2-[(18)F]fluoro-2-deoxyglucose (FDG) avid, defines “radioiodine-refractory” DTC (RR-DTC) (7). Poorly differentiated thyroid carcinoma (PDTC) and anaplastic thyroid carcinoma (ATC) are rare, accounting for 3–5% and 1% of all thyroid cancers, respectively, and are the most aggressive follicular cell-derived thyroid cancer. Poorly DTC and ATC generally do not take up RAI, may not secrete Tg or respond to thyroid-stimulating hormone (TSH) (8). Poorly DTC should be histologically distinguished from well-differentiated papillary and follicular cancers (5). Despite the low incidence, PDTC accounts for a disproportionate number of thyroid cancer-related deaths because of its resistance to the most common therapeutic approaches. Medullary thyroid cancer (MTC) originates from the para-follicular C-cells of the thyroid accounts for 1–2% of thyroid cancer, but will not be discussed here. This review will describe the current treatment of locally A-DTC, metastatic DTC and RR-DTC (Figure 1).

## Locally A-DTC

Extensive ETE is rare, reported to occur in 4–8% of patients with DTC at the time of diagnosis (3, 9). These patients are classified as pT4 using the AJCC/UICC TNM system, defining T4a as a tumor of any size extending beyond the thyroid capsule to invade subcutaneous soft tissues, larynx, trachea, esophagus, or recurrent laryngeal nerve, and T4b as tumor invading prevertebral fascia or encasing the carotid artery or mediastinal vessels (10). Contrast enhanced CT, MRI, and endoscopy (to specifically assess endo-luminal involvement) are useful investigations to accurately assess extent of disease and avoid under-staging, thereby informing the correct management decisions (11). Surgery is the primary treatment modality of choice for A-DTC, to resect all gross disease while minimizing morbidity. A dedicated thyroid surgeon with a high-volume practice is critical to achieve this. A more conservative approach may be considered in patients with minimal visceral invasion, allowing “shave procedures” to remove macroscopic disease while accepting there will be residual microscopic disease, which may be managed with adjuvant RAI (12). In the event of transmural invasion of the esophagus, trachea or larynx a full thickness resection is recommended if possible. When the invasion is limited to the superficial layers, there is no consensus as to the extent of surgery, with some groups recommending an aggressive approach and others a more conservative approach with adjuvant therapy. For patients with distant metastases, although cure is unlikely to be achieved, there is still a role for surgery. Total thyroidectomy, if possible, allows subsequent treatment of metastatic disease with RAI. Palliative resection of bulky disease may reduce distressing symptoms such as airway compromise or hemorrhage. External beam radiotherapy (EBRT) may also palliate symptoms. A personalized approach should be applied to the patient and preferably discussed within a multi-disciplinary setting. RAI is recommended for ATA high risk patients after total thyroidectomy with the purpose of not only ablating normal remnant but also as an adjuvant treatment for residual microscopic disease (5). Subsequent therapy doses may be required if ablation does not result in complete response. EBRT may be considered adjuvantly in selected patients with aerodigestive tract invasion or where there is gross residual disease (R2 resection). The role and indications for EBRT are controversial as there is no good prospective data to guide decision-making. Available evidence suggests an advantage in terms of local control in patients with R2 resections (13, 14). RAI ablation should also be administered either before or after EBRT; the optimal sequencing is unknown although there is concern that the remnant and disease may be less RAI avid post EBRT. Initial TSH suppression is recommended at less than 0.1 μU/L, and then adjusted according to response to therapy (5). The response to initial therapy is prognostic; patients who have residual structural disease have a poorer outcome than those with a biochemical incomplete response (persistent Tg but no structural disease).

## Metastatic DTC

Patients with distant metastases have a reasonable life expectancy with survival measured in years. One study of patients with lung metastases reports a median overall survival (OS) of 10.45 years and a median progression-free survival (PFS) of 3.65 years (15). Even better outcomes have been described in younger patients with low disease burden (6). Several clinical factors have been reported to confer poorer outcome including older age, high FDG avidity, aggressive tumor histology, poor RAI avidity, and initial high stage (16). The most frequent sites of distant metastases are lung, bone, brain, liver and skin (6), and multiorgan metastases are associated with a poorer survival compared to single-organ metastases (17). Treatment for metastatic disease includes TSH suppression (TSH < 0.1 μU/L) and RAI therapy while the disease remains iodine avid and sensitive. RAI is the first line and a very effective treatment for distant metastases from papillary and follicular carcinoma (18). Two-thirds of patients with distant metastases will have disease that is RAI avid. Treatments every 6–12 months depending on rate of growth and response can be considered. About one-third of patients will achieve remission (i.e., negative imaging), after a median cumulative 131-I activity of 8.1 GBq (220 mCi) (6). There is no specific upper limit of RAI treatment but once the disease stops taking up RAI, progresses in spite of RAI or new non-avid disease appears, then there is no benefit with further treatments and alternative therapies should be considered. Despite seventy years of medical use of RAI, there are still controversies on the optimal therapeutic activity (19). There are two main approaches to RAI therapy: an empiric (administration of standard activities) or a dosimetry-based (prescribing to predicted absorbed radiation dose) approach. OS between the two different approaches has been evaluated although only in a retrospective review comparing patients treated by an empiric fixed activity of 3.7 GBq in one center with those treated by personalized activity (2.7–18.6 GBq) based on whole-body/blood clearance (WB/BC) dosimetry in another. There was no OS advantage for the dosimetric approach; however, there were inherent problems with such a retrospective review (20) and clearly prospective randomized data is required to fully answer this question. Locally ablative treatments such as surgery, radiofrequency ablation (RFA), cryoablation, EBRT, chemo-embolization, and ethanol ablation can be used in selected patients for specific areas of problematic disease. Most of the available data regarding focused locally ablative therapy have been obtained from non-thyroid cancer patients and the standard of care can vary significantly between different cancer centers. In selected patients, local treatment modalities such as stereotactic radiation therapy (SBRT) and percutaneous ablation may be as effective as surgery to induce local tumor control and could represent a first-line treatment. These modalities can be useful in improving symptom control and delaying the initiation of systemic treatment or during systemic therapy where targeting progression in a single lesion may enable continued overall control of disease (5). RFA or SBRT may afford for local control in solitary lung metastases. RFA is a well-tolerated treatment that showed high efficacy in clinical trials of lung metastases from other solid tumors, with a complete response in 88–93% of patients with after 12–18 months and local control rates ranging from 63 to 98% (21–23). There are no data on the efficacy in thyroid cancer specifically. The most common site of bone metastases is the spine, frequently associated with neurological deficit, reduced quality of life due to pain, and increased mortality (24). Treatment options include surgical resection, vertebroplasty, EBRT (including SBRT), and RFA. These local treatments are indicated for symptomatic or imminently symptomatic disease, even in the presence of RAI uptake (25). Seventy percent of patients with metastatic DTC involving bones developed at least one skeletal-related event: spinal cord compression, pathological fracture, requiring EBRT or surgery or malignant hypercalcemia (26). Bisphosphonate or denosumab therapy should be considered in patients with symptomatic and/or diffuse bone metastases. These agents delay time to occurrence of events and improve symptoms (27). Three-monthly versus monthly intravenous infusion of bisphosphonates is still a matter of debate and randomized trial data are needed. Brain metastases usually occur in older patients and are associated with poor prognosis. Neurosurgical resection, SBRT, or whole brain radiotherapy are the main treatments (28, 29). Whole brain RT should be considered with multiple widely spread metastases but SBRT can be used for a low number of small volume deposits in patients with a good performance status. RAI therapy may be effective, with concomitant glucocorticoid therapy prior to treatment to minimize edema and subsequent neurological deterioration. RFA represents a useful treatment modality in patients with liver metastases replacing techniques such as cryoablation, due to better efficacy, lower complication rate, and lower incidence of tumor recurrence (30). In highly vascularized tumors, RFA may be preceded by selective transarterial embolization, because the decrease in blood supply reduces local heat dissipation and allows tumor necrosis (31). RFA of hepatic metastases achieves local control rates ranging from 40 to 80% (32, 33). The morbidity rate of RFA for hepatic lesions (both primary tumors and metastases) is reported to be 4–9%, with a mortality rate below 1% (34, 35). SBRT has also been reported to produce local control rates ranging from 57 to 100% in liver metastases (23).

## RAI Refractory DTC

Limited treatment options are available for patients with progressive and RR-DTC. Conventional chemotherapy has limited efficacy and significant toxicities. Doxorubicin remains the most effective conventional agent, 60–75 mg/m^2^ every 3–4 weeks, but has poor response rates (36). A greater understanding of the biology of thyroid cancer has led to the development of “targeted” therapies. Multiple pathways are involved in tumor angiogenesis, growth, and progression. Alongside, this understanding of the molecular background to the evolution of thyroid cancer, agents have been developed to block these inappropriately activated pathways within the cancer cells. Two agents, Sorafenib and Lenvatinib, have been approved by the US Food and Drug Administration and European Medicines Agency, based on data from multi-center, randomized, double-blind, placebo-controlled phase III studies, the DECISION and SELECT trials, respectively, showing a benefit in terms of PFS over placebo. Sorafenib is a multi-tyrosine kinase inhibitor (MKI), which shows inhibitory activity against the vascular endothelial growth factor receptors (VEGFR) 1, 2, and 3, platelet-derived growth factor receptor (PDGFR) β, Raf-1, RET, and BRAF (37). In the phase III study, the median PFS in patients treated with Sorafenib was significantly improved compared to the placebo group (10.8 versus 5.8 months). Lenvatinib is a MKI of the VEGFRs 1, 2, and 3, FGFRs 1 to 4, PDGFR α, RET, and KIT signaling networks, which significantly improved median PFS compared to placebo in the phase III study (18.3 versus 3.6 months) (38). MKIs should be started in patients with progression of measurable lesions, as defined radiologically by RECIST criteria, over the previous 12–14 months and must take into consideration tumor burden, site of the lesions, symptoms, and the risk of local complications (7, 39). The optimal time to start therapy with an MKI is still a matter of debate, especially for asymptomatic patients. An international, prospective, open-label, multicentre, non-interventional study (RIFTOS MKI study) is investigating the time to symptomatic progression from study entry in asymptomatic patients with progressive RR-DTC, with 700 patients estimated to be enrolled in over 20 countries (Clinical trial: 02303444) (40). Sabra et al. reported that average tumor volume doubling time of lung metastases could be used to predict eligibility for systemic therapy with MKIs, but this has to be confirmed with further data (41). Although these drugs represent a promising option for the treatment of RR-DTC, they are associated with significant side effects including most commonly hypertension, diarrhea, hand/foot skin reactions, rash, fatigue, mucositis, loss of appetite, and weight loss (Table 1). An important adverse event of some kinase inhibitors can be prolongation of QT interval on electrocardiograms (ECGs), which if unchecked and unresolved lead to the risk of Torsade de Pointes and other arrhythmias. Although this AE is not commonly reported with either sorafenib or lenvatinib (it is seen with vandetanib licensed for advanced MTC), ECGs should be carried out at baseline and at each follow-up to monitor this and interruption of the drug and cardiology review should be sought. Many commonly used drugs cause QT prolongation, so, a regularly updated review of a patient’s concomitant medications must be part of the assessment. The impact of these adverse events (AEs) on quality of life versus any potential benefit for the patient must be carefully evaluated. Most AEs will occur within the first few weeks of treatment. It is, therefore, recommended that patients are reviewed every 7–15 days for the first month in order to detect toxicities early and intervene with supportive medication, e.g., antihypertensives, antidiarrhoeals, and advice such as skin care. If these methods are not successful, drug dose reduction or interruption may be required (42, 43). Subsequently, monthly visits and then less frequently once a tolerable and effective dose is established. Monitoring of affective disorders is also important and these drugs can lower mood and at worst lead to suicidal ideation. Prompt management of AEs is fundamental to maintaining dose intensity and quality of life on these drugs (44). An area of active research is the attempt to reverse the refractoriness for RAI by trying to re-establish uptake by thyroid cancer cells. A single-center small trial has tested a MEK inhibitor, Selumetinib, in patients with RR-DTC, with promising results in a subgroup of patients. An increase in uptake on iodine-124 PET/CT in 12 out of 20 patients reaching an arbitrary dosimetry threshold for RAI therapy in 8 patients (45). While these results are promising, questions remain unanswered and currently the SEL-I-METRY trial (EudraCT no. 2015-002269-47) in United Kingdom is trying build on this preliminary data in RR-DTC (46).

**Table 1:** Most common toxicities for the two licensed multi-tyrosine kinase inhibitors reported in clinical trials (more than 5%).

Sorafenib (*N* = 207)			Lenvatinib (*N* = 261)		
	All grades (%)	Grade ≥3 (%)		All grades (%)	Grade ≥ 3 (%)
Hand–foot skin reaction	76.3	20.3	Hypertension	67.8	41.8
Diarrhea	68.6	5.3	Serum thyroid-stimulating hormone (TSH) increase^a^	61.5	–
Alopecia	67.1	–	Diarrhea	59.4	8
Rash/desquamation	50.2	4.8	Fatigue	59.0	9.2
Fatigue	49.8	5.8	Anorexia	50.2	5.4
Weight loss	46.9	5.8	Weight loss	46.4	9.6
Hypertension	40.6	9.7	Nausea	41.0	2.3
Serum TSH increase^a^	33.3	-	Stomatitis	35.6	4.2
Anorexia	31.9	2.4	Hand–foot skin reaction	31.8	3.4
Oral mucositis	23.2	1	Proteinuria	31	10
Pruritus	21.3	1	Vomiting	28.4	1.9
Nausea	20.8	0	Headache	27.6	2.7
Headache	17.9	0	Dysphonia	24.1	1.1
Cough	15.5	0	Arthralgia	18	0
Constipation	15	0	Dysgeusia	16.9	0
Dyspnea	14.5	4.8	Rash	16.1	0.4
Neuropathy (sensory)	14.5	1	Constipation	14.6	0.4
Abdominal pain	14	1.4	Myalgia	14.6	1.5
Pain (extremity)	13.5	0.5	Dry mouth	13.8	0.4
Dermatology (other)	13	1	Upper abdominal pain	13	0
Voice changes	12.1	0.5	Abdominal pain	11.5	0.4
Fever	11.1	1.5	Peripheral edema	11.1	0.4
Vomiting	11.1	0.5	Alopecia	11.1	0
Back pain	10.6	1	Dyspepsia	10	0
Pain (other)	10.6	0.5	Oropharyngeal pain	10	0.4
Pain (throat, pharynx, larynx)	10.1	0	QTc prolungation	8	1.5
Hypocalcemia	18.8	9.2	Hypocalcemia	6.9	2.7
Increased ALT	12.6	2.9	Arterial thromboembolic effects	5.4	2.7
Increased AST	11.1	1			

*^a^More than 0.5 μIU/L*.

*Adapted from Ref. (37, 38)*.

## Current Issues in Advanced Thyroid Cancer

The 2015 ATA guidelines encourage a less aggressive approach toward DTC, for example, supporting lobectomy instead of total thyroidectomy for low-risk DTC, reducing thyroid hormone suppression based upon risk of disease recurrence and revaluating the impact of microscopic ETE (5, 47). Active surveillance has been proposed for papillary micro-carcinoma, after initial studies from Japan that have shown that immediate surgery and watchful waiting are equally effective in avoiding deaths from thyroid cancer (48). In an observational study of 291 patients in the United States, DTC ≤1.5 cm were actively observed and growth in tumor diameter of 3 mm or more was found in only 3.8% of patients, suggesting that this approach might be reasonable (49).

The impact of a less aggressive approach on A-DTC is not known. The dramatic increase in the incidence of DTC is probably due to increased medical surveillance and most of these DTC were small, low-risk papillary carcinomas where a large fraction could represent over-diagnosis, with unnecessary surgery and potential other harmful cancer treatments (50). However, the SEER data also show an increase of thyroid cancer mortality of 1.1% per year from 1994 to 2013 overall and 2.9% for metastatic DTC (51). Despite the paradigm shift toward reduced intensity treatment for DTC, A-DTC may cause significant symptoms and shortening of life and should be managed proactively. The search to find predictive and prognostic biomarkers to identify which patients will develop A-DTC continues.

## Future Perspectives

Significant progress has been made in the management of A-DTC. The approval of two drugs, Sorafenib and Lenvatinib, following phase 3 data showing delayed time to disease progression compared to placebo means there are now treatment options for RR-DTC. Identification of patients who will not respond to initial treatment and go on to develop A-DTC remains an area of active research. Our understanding of the molecular basis of papillary thyroid carcinoma (PTC) has been greatly enhanced following an extensive genomic analysis completed as part of the cancer genome atlas. This led to the identification of the driver genetic alterations in more than 90% of PTC, with broad implications for diagnosis, prognosis, and therapeutic targets. Indeed, molecular signatures are expected to have a significant impact on therapeutic decisions in all forms of DTC, PDTC, and ATC, perhaps guiding clinicians as to which groups of patients warrant treatment, which treatment is likely to be the most effective, thereby providing a truly personalized approach. With this targeted approach, promising results have been obtained in BRAF-mutated PTC with the selective BRAF inhibitors dabrafenib and vemurafenib (52, 53). Treatments directed at the VEGFR including axitinib, pazopanib, motesanib, and sunitinib have also shown activity in A-DTC (54–57). Finally, immunotherapy is being explored in A-DTC. Pembrolizumab, a checkpoint inhibitor against the programmed death protein-1, is currently being tested in combination with lenvatinib or as second line after progression on lenvatinib (https://ClinicalTrials.gov Identifier: NCT02973997).

## Conclusion

In the past decade, advances in the treatment of DTC have been achieved. Surgery, TSH suppression and RAI remain the most effective modalities for patients at higher risk for disease recurrence or mortality. Advances in assessment and management of A-DTC have led to excellent survival outcomes. Targeted therapies, specifically MKIs, are changing the therapeutic landscape for patients with RR-DTC. Active areas of research include the definition of the optimal time of initiation systemic therapy, molecular characterization to refine treatment decisions and the possible efficacy of immunotherapy.

## Author Contributions

DT, FF, and KN have made a substantial, direct, and intellectual contribution to the work and approved it for publication.

## Conflict of Interest Statement

KN has had advisory and speakers’ bureau roles with Astra-Zeneca, Eisai, and Sanofi-Genzyme. All other authors declare that the research was conducted in the absence of any commercial or financial relationships that could be construed as a potential conflict of interest.
